# Pyoderma gangrenosum, peripheral ulcerative keratitis, and oral ulcers in a patient with inflammatory bowel disease

**DOI:** 10.1016/j.jdcr.2025.04.013

**Published:** 2025-04-26

**Authors:** Amanda T. Chung, Roy Luister C. Acos, John Paulo L. Recio, Giselle Marie S. Tioleco-Ver, Belen L. Dofitas

**Affiliations:** a Department of Dermatology, University of the Philippines - Philippine General Hospital, Manila, Philippines; b Section of Dermatology, Department of Internal Medicine, Adela Serra Ty Memorial Medical Center, Tandag, Philippines; c Department of Medicine, University of the Philippines - Philippine General Hospital, Manila, Philippines

**Keywords:** inflammatory bowel disease, oral ulcers, peripheral ulcerative keratitis, pyoderma gangrenosum

## Introduction

Pyoderma gangrenosum (PG) is an inflammatory neutrophilic dermatosis that presents as tender papules, pustules, or vesicles that rapidly ulcerate, often affecting the legs.^1^ It is a debilitating extraintestinal manifestation (EIM) of inflammatory bowel disease (IBD), occurring in 0.5% to 5% of cases.^2^

Peripheral ulcerative keratitis (PUK) is a corneal inflammatory disease characterized by a crescent-shaped epithelial defect, stromal thinning, and infiltration. Nearly half of noninfectious PUK cases are associated with autoimmune conditions, including PG and IBD. Without timely intervention, PUK can progress to corneal perforation and potential blindness.^3^^,^^4^

Both PUK and oral ulcers have been associated with PG and IBD.^3^^,^^5^, ^6^, ^7^ This report details a rare case of concurrent PG, PUK, and oral ulcers in a 49-year-old woman with IBD.

## Case report

A 49-year-old woman presented with a 2-month history of necrotic ulcers on the trunk and lower extremities, initially appearing as violaceous bullae on the right foot. After debridement at a local hospital, she developed multiple ulcers on the trunk and legs, associated with intermittent fever, left eye pain with ocular discharge, photophobia, and oral ulcers.

Seven months prior, she was admitted for lower gastrointestinal bleeding, with endoscopic findings of erosive duodenopathy, ileitis, and nonspecific colitis. Colonic biopsy showed cryptitis, crypt abscess, and crypt rupture. Postdischarge, she had no gastrointestinal complaints.

**Fig 1:**
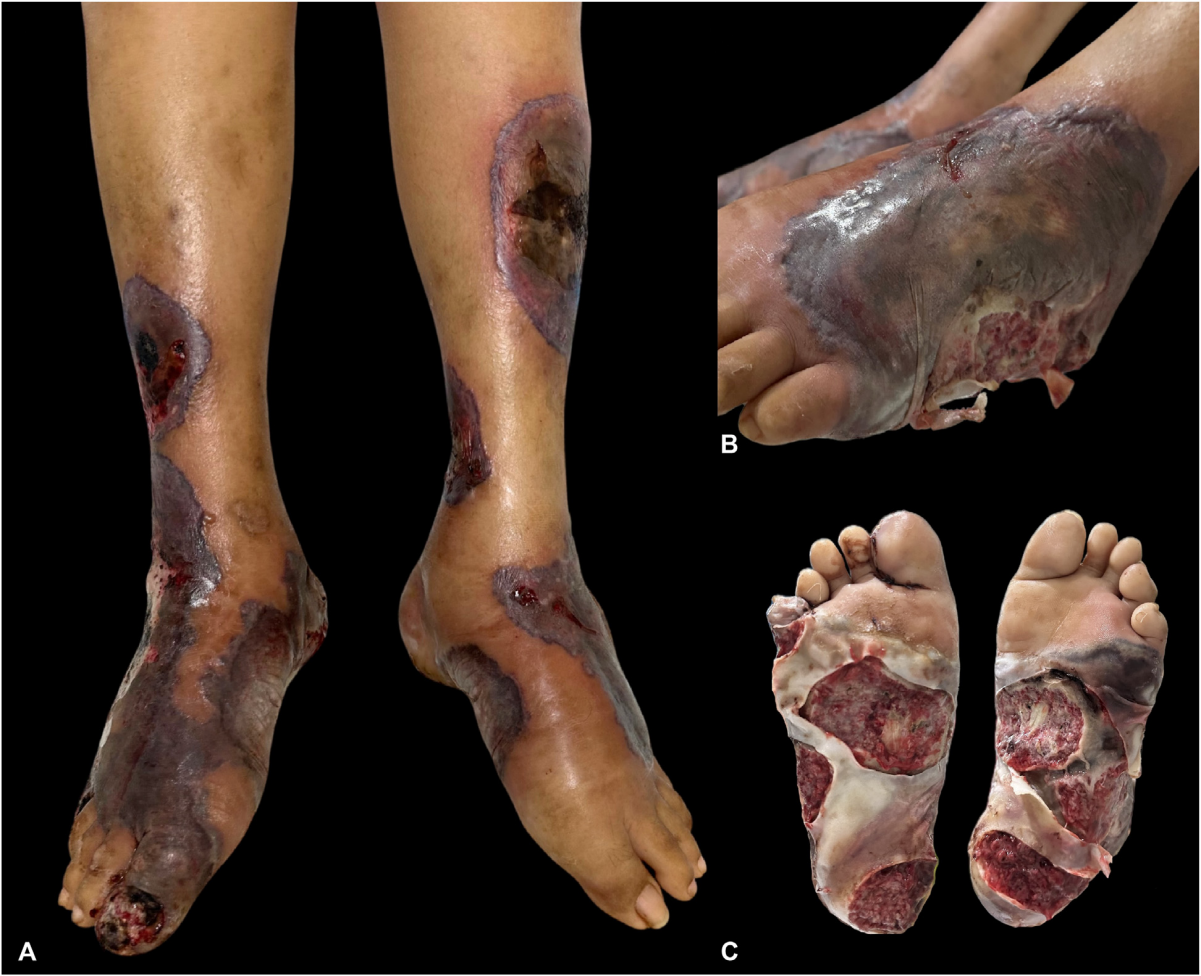
Cutaneous findings. Multiple large, necrotic, hemorrhagic ulcers with violaceous and bullous borders on (**A**) legs, (**B**) dorsum of left foot, and (**C**) bilateral soles.

**Fig 2:**
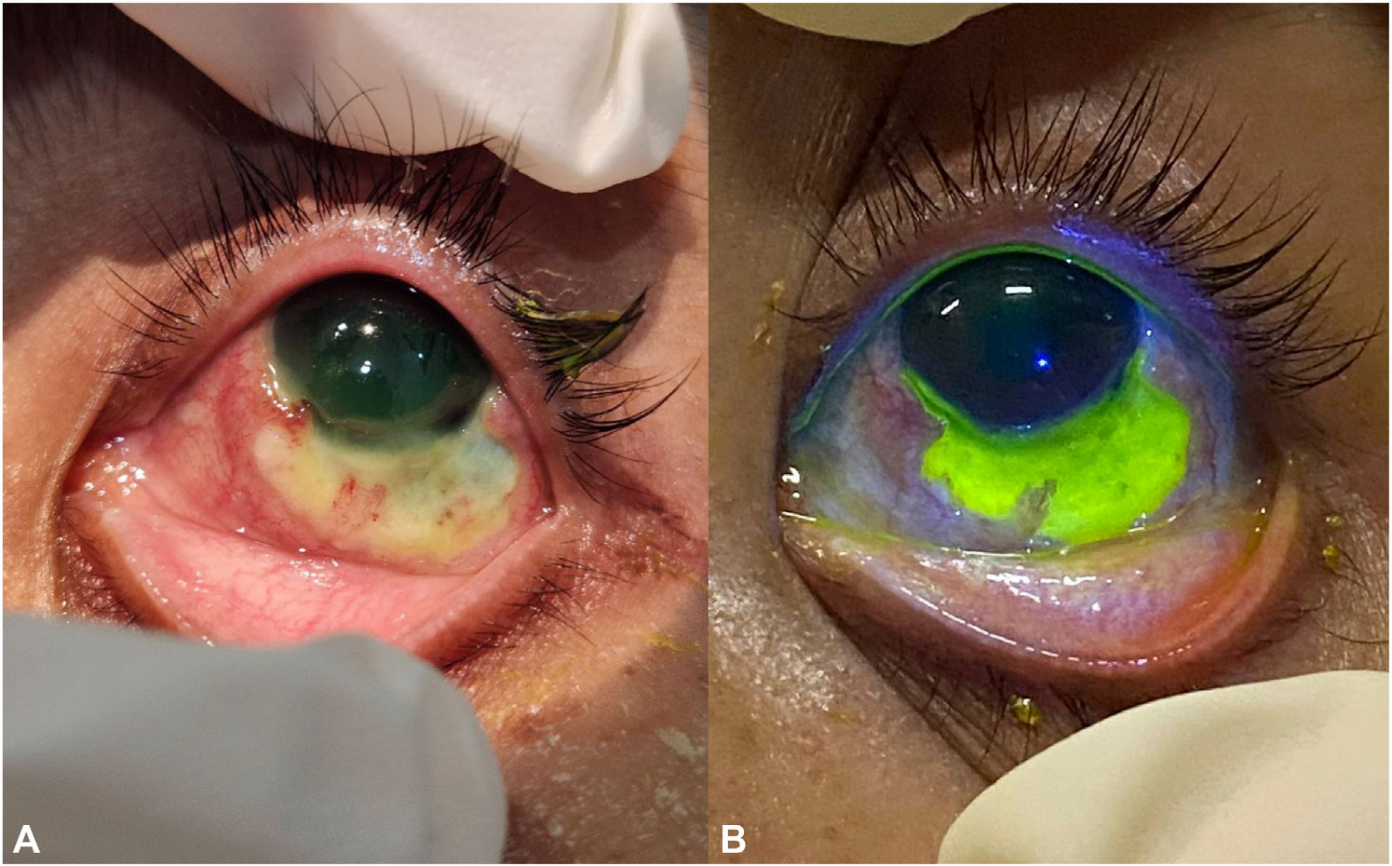
Peripheral ulcerative keratitis. **A,** 1.5 × 10 mm crescent-shaped, epithelial-stromal defect from 3 to 8 o’clock at the inferior perilimbal area. **B,** Inferior to the corneal defect, there was a 4 × 10 mm bulbar conjunctival defect from 4 to 7 o’clock exposing an area of avascularized sclere, with pooling of fluorescein dye.

**Fig 3:**
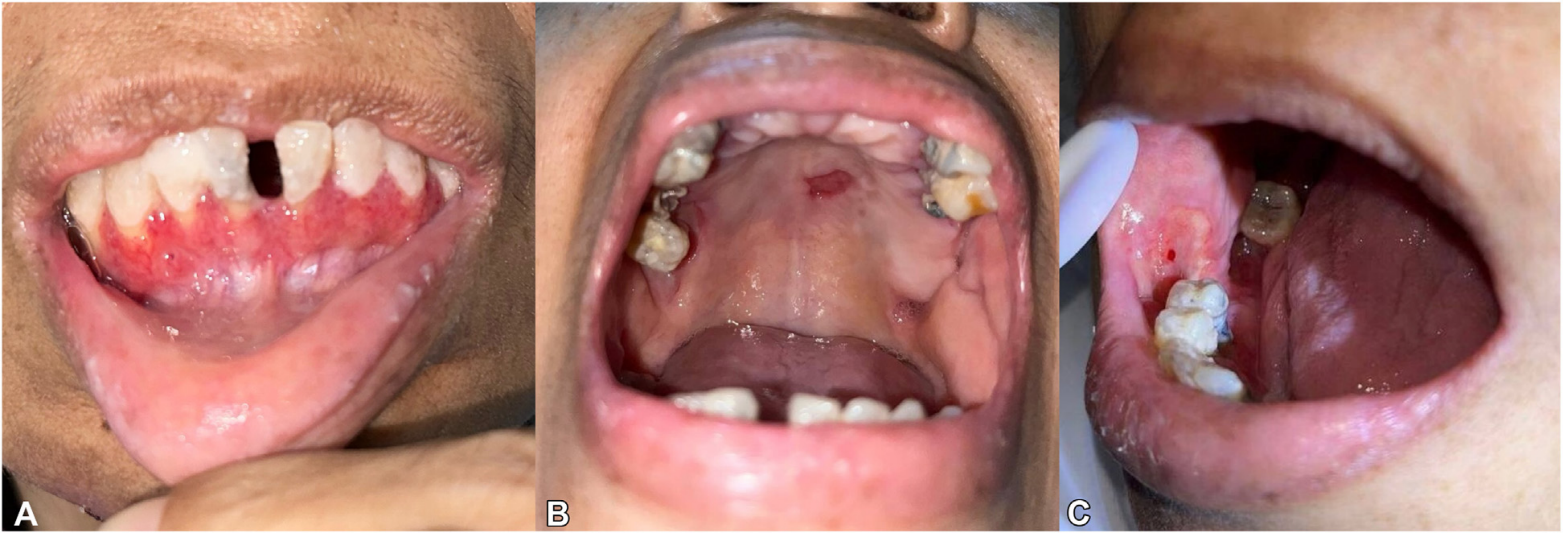
Oral ulcers. **A,** Erythematous ulcers on the labial gingiva. **B,** Solitary, irregularly shaped ulcer, with beefy red base and rolled borders, measuring 1.5 × 0.6 cm, on the hard palate. **C,** Solitary, irregularly shaped ulcer, with fibrinous base and pinpoint bleeding, measuring 1.8 × 1.8 cm, on the right buccal mucosa.

Examination revealed multiple necrotic ulcers with violaceous borders on the dorsum and soles of bilateral feet extending to the legs (Fig 1, *A*). Similar lesions were noted on the dorsum of the right hand, left breast, umbilicus, and sacral region. Ophthalmologic examination showed a 1.5 × 10 mm crescent-shaped epithelial-stromal defect in the cornea, along with a 4 × 10 mm bulbar conjunctival defect exposing avascular sclera (Fig 2, *A* and *B*). Intraoral examination revealed multiple ulcers with reddish base on the labial gingiva, hard palate, and buccal mucosa (Fig 3, *A*-*C*). No lymphadenopathies were palpated.

**Fig 4:**
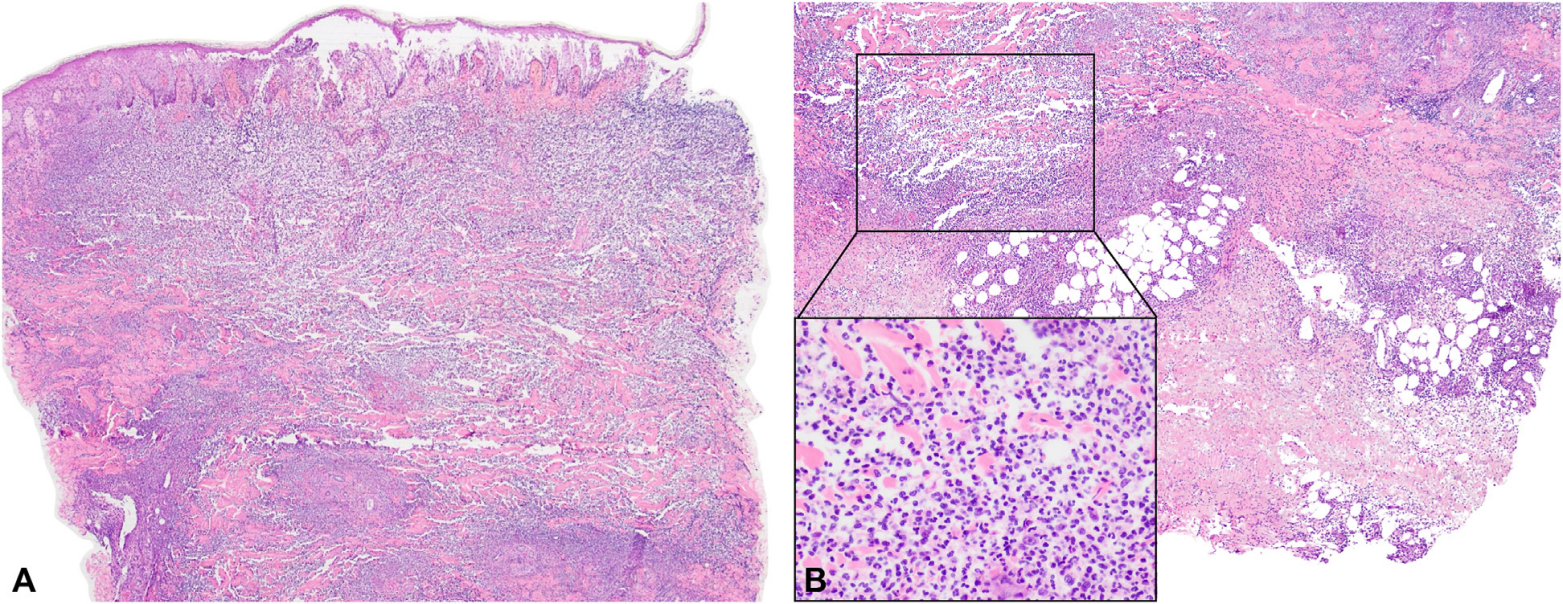
Histopathologic findings. Punch biopsy taken from the border of left leg ulcer showing an intraepidermal blister, dense perivascular and interstitial infiltrates composed of predominantly neutrophils and neutrophilic nuclear dusts (inset) in the superficial to deep dermis, extending to the subcutis. **A,** H&E, ×4. **B,** H&E, ×10 (H&E, ×100 [inset]). *H&E*, Hematoxylin and eosin.

Skin biopsy of the left leg ulcer showed neutrophilic dermatitis, consistent with PG (Fig 4, *A* and *B*). Blood tests showed anemia, leukocytosis with neutrophilic predominance, and positive perinuclear neutrophil antibodies (5.1; <3.5 negative). Peripheral blood smear was unremarkable. Colonoscopy was unremarkable. Representative biopsies of the colonic mucosa showed chronic nonspecific inflammation. Qualitative fecal calprotectin was positive. Blood and tissue cultures, chest and abdominal contrast-enhanced tomography, and arteriovenous duplex scan were unremarkable.

The patient was diagnosed with PG, PUK, and mild IBD. Treatment began with intravenous hydrocortisone 100 mg intravenous every 12 hours and doxycycline 100 mg/cap 1 cap every 12 hours, later transitioning to methylprednisolone pulse therapy (500 mg/day) for 3 days. Ulcers were dressed with a rotation of silver-impregnated hydrofiber and povidone-iodine-impregnated foam. Ocular and oral treatments included sodium hyaluronate, levofloxacin, prednisolone acetate eye drops, and prednisone-lidocaine mouthwash. Despite immunosuppressive therapy, ulceration progressed, prompting infliximab infusion (5 mg/kg) on day 17. This halted ulcer progression, led to conjunctival re-epithelialization, and resolved oral ulcers within 4 days. Further infliximab doses were administered at weeks 2 and 6. The patient was discharged on day 37, but developed contractures due to prolonged immobilization, rendering the patient wheelchair-bound.

Over the next 3 months, ulcers continued to re-epithelialize and granulate. However, she was readmitted to a local hospital for severe anemia requiring blood transfusion and was lost to follow-up at our institution. We were later informed that she expired at home due to sepsis.

## Discussion

The diagnosis of PG met the Delphi consensus criteria (1 major and 6 minor).^1^ Given its similarity to infections, vasculitis, and vascular ulcers, and its potential malignancy association, a thorough work-up was performed to rule out these conditions. Given colitis on endoscopic and biopsy findings 7 months prior and the absence of gastrointestinal symptoms during the current admission, mild IBD was considered the most likely PG trigger.

EIMs of IBD can be classified by their relationship to intestinal disease activity. Some, like erythema nodosum and oral aphthous ulcers, parallel IBD flares, while others, such as uveitis and ankylosing spondylitis, progress independently. PG and PUK fall into a third category with an unclear association with IBD activity.^8^ Ocular manifestations of IBD are common, particularly anterior uveitis and episcleritis.^9^ PUK is rare but has been reported in IBD cases requiring immunosuppressive therapy.^3^ Oral manifestations occur in up to 37% of adult IBD patients, with aphthous stomatitis being the most common. Other manifestations include pyostomatitis vegetans, mucosal cobblestoning, and orofacial granulomatosis.^9^ Most of these oral manifestations follow the activity of gut disease, making them useful clinical markers for IBD exacerbation.^8^

PG has been implicated as a cause of PUK and oral ulcers, particularly in the presence of IBD.^5^, ^6^, ^7^ The most common presenting sign of ocular PG is ulceration, PUK, and decreased visual acuity.^5^ Oral ulcers associated with PG are irregular, with necrotic or friable bases.^6^ Differentiating PG versus IBD-related mucosal lesions is challenging due to similar presentation and nonspecific biopsy findings. However, in this case, the timing of mucosal lesions paralleling PG rather than IBD activity suggests they were manifestations of PG. Moreover, PUK is more consistent with PG. Paraneoplastic syndromes can also occur and present similarly, but there was no evidence of hematologic or solid organ malignancy on work-up.

The first-line treatment for severe, refractory cases of PG is intravenous corticosteroids and tumor necrosis factor alpha antagonists.^9^ Infliximab is an effective treatment for IBD-associated PG,^10^ which the patient’s cutaneous, ocular, and oral manifestations responded to.

Distinguishing between IBD-related EIMs and PG mucosal lesions is clinically significant as it can potentially influence treatment decisions. While both conditions respond to systemic immunosuppression, ocular and oral PG may indicate a more severe, refractory disease course, warranting close monitoring and aggressive treatment. Recognizing and managing such cases requires a multidisciplinary approach involving ophthalmologists, dentists, and gastroenterologists.

## Conflicts of interest

None disclosed.
